# Concomitant Septal Myectomy with Aortic Valve Replacement for Severe Aortic Stenosis with Left Ventricular Outflow Tract Obstruction

**DOI:** 10.14789/jmj.JMJ22-0036-OA

**Published:** 2023-05-20

**Authors:** AKIKO UMETSU, SATOSHI MATSUSHITA, TAKESHI KINOSHITA, MINORU TABATA

**Affiliations:** 1 Department of Cardiovascular Surgery, Juntendo University Graduate School of Medicine, Tokyo, Japan; 1 Department of Cardiovascular Surgery, Juntendo University Graduate School of Medicine, Tokyo, Japan

**Keywords:** left ventricular outflow tract obstruction, aortic stenosis, myectomy, aortic valve replacement, hypertrophic cardiomyopathy

## Abstract

**Objectives:**

Septal myectomy confers survival benefits on patients with hypertrophic cardiomyopathy. However, its role in the treatment of severe aortic stenosis (sAS) with left ventricular outflow tract obstruction (LVOTO) remains under investigation. Another challenging question in the era of transcatheter aortic valve replacement is who would benefit more from traditional surgical aortic valve replacement (SAVR) with myectomy. Therefore, this study aimed to investigate myectomy cases at our hospital in Japan.

**Methods:**

A total of 740 patients who underwent SAVR for sAS between 2012 and 2019 were identified. The demographics and baseline echocardiographic findings were retrospectively compared between patients who underwent concomitant myectomy and those who did not. The myectomy group was further assessed for factors predisposing to LVOTO, operative details, echocardiographic changes, and prognosis. The resected septa were histopathologically analyzed.

**Results:**

The myectomy group mostly comprised elderly females with a small hypercontractile heart. Myectomy with SAVR led to statistically significant improvements in concentric left ventricular hypertrophy and LVOTO parameters. Survival was comparable with that reported in previous reports, even in the elderly subset (≥ 75 years). The septa showed mild fibrosis.

**Conclusions:**

Myectomy can be safely performed with SAVR for sAS with LVOTO, even in the elderly, and it effectively improves LVOTO. Special attention should be paid to elderly females with relatively more severe AS and a small yet extra-hypertrophic and extra-hypercontractile heart. Such patients warrant comprehensive assessment of LVOTO, and despite its invasiveness, SAVR may be potentially more beneficial by allowing direct observation of LVOTO and ancillary myectomy.

## Introduction

Septal myectomy is an open-heart procedure wherein part of the interventricular septum (IVS) is removed when it is associated with left ventricular outflow tract (LVOT) obstruction (LVOTO)^1)^. Classically, LVOTO accompanies the obstructive subtype of hypertrophic cardiomyopathy (HCM)^2)^. Therefore, myectomy has been performed on patients with HCM and recommended as the gold standard treatment for them, as it improves long-term survival^3, 4)^. More recently, LVOTO has been recognized as a manifestation of a wider array of disease entities such as aortic stenosis (AS), left ventricular hypertrophy (LVH), asymmetric septal hypertrophy (ASH), and systolic anterior motion of the mitral valve (SAM)^5, 6)^. It has also been reportedly associated with various conditions of acutely reduced pre- or afterload under severely hyper- or hypokinetic left ventricle (LV)^7, 8)^ and may appear as treatment-resistant shock that paradoxically worsens upon ionotropic support^9, 10)^. These findings may indicate that more conditions may reap the benefits of septal reduction therapy.

Just as myectomy is recommended for patients with LVOTO, aortic valve replacement (AVR) has been recommended as the gold standard treatment for patients with severe AS (sAS)^11)^ because it improves long-term survival^12)^. Historically, myectomy has been concomitantly performed with surgical AVR (SAVR) on patients with sAS who are at risk of LVOTO^13, 14)^, as latent LVOTO that has been masked by sAS in a state of dual obstruction of LV can be unmasked after AVR, or LVOTO can develop *de novo*^9, 10)^. For example, with the increasing use of transcatheter AVR (TAVR) for sAS, more studies have reported sudden incidence and worsening of LVOTO immediately after TAVR in an event called “suicide LV^15, 16)^,” which may have been prevented if risks were known beforehand.

Some authors have suggested that intraoperative decisions for myectomy are critical in patients at risk of LVOTO. Kayalar *et al*. analyzed cases of concomitant myectomy with SAVR for sAS and reported that myectomy is safe and effective and that 72% of decisions for myectomy were intraoperative. Therefore, they proposed that myectomy be considered in the setting of ASH even if the obstruction has not been previously demonstrated^17)^. Similarly, Lim *et al*. reported that an intraoperative finding of ASH was common during SAVR for sAS and that myectomy was performed in these patients without any additional risks^18)^. Although the prevalence of LVOTO is currently unknown, consequences of overt LVOTO are concerning, from perplexing manifestations in the acute phase to need for repeat surgery in the chronic phase^19)^. Therefore, overt LVOTO must be treated, and latent LVOTO should be detected.

This study aimed to characterize factors predisposing to LVOTO and investigate the safety and effectiveness of myectomy in patients with sAS and LVOTO, to better serve this unique patient group that may particularly benefit from myectomy with SAVR.

## Patients and Methods

### Patients

The study was approved by the Institutional Review Board of Juntendo University Hospital (approval # E22-0301). The requirement of informed consent was waived due to general consent obtained at the time of admission and the retrospective observational nature of the study. A total of 740 patients who underwent SAVR for sAS between 2012 and 2019 were identified from our consecutive patient list. SAS was defined as an aortic valve (AV) area (AVA) ≤ 1.0 cm^2^, AV systolic mean pressure gradient (mPG) ≥ 40 mmHg, or AV peak velocity ≥ 4.0 m/s on preoperative transthoracic echocardiography (TTE). Cases of combined and repeat surgeries were included.

### Clinical data collection and analysis

Patient details were collected from a review of medical records. Overall, 68 patients were excluded due to partial TTE reports. One patient was excluded because of a preoperative complication of infective endocarditis. The remaining 671 patients were divided into the following two groups: myectomy group that underwent SAVR with myectomy and AVR group that underwent SAVR without myectomy. The demographics, basic physical characteristics, and baseline TTE findings were compared between the two groups, followed by a single-arm cohort study of the myectomy group for comorbidities, operative details, TTE changes, complications, and prognosis.

Before an intergroup comparison, each TTE parameter was compared with its reference value for each sex, as proposed by the American Society of Echocardiography and the European Association of Cardiovascular Imaging^20)^. Patients from each group were first classified into female and male subgroups, and the respective reference value was subtracted from each measurement. The results were combined and compared against zero, and *P*-value was calculated using paired Student's *t*-test as described in the Statistical Analysis section below.

Relative wall thickness (RWT), LV mass (LVM), and LV mass index (LVMI) were calculated following society recommendations^20)^. Since LVOTO may arise from undiagnosed HCM or ASH, frequencies of positive echocardiographic criteria, i.e., IVS or posterior wall (PW) ≥ 15 mm for HCM, and IVS- to-PW ratio (IVS/PW) > 1.3 for ASH, were additionally analyzed with frequencies of sigmoid septum and SAM^21, 22)^. In this study, a high LVOT systolic peak pressure gradient (pPG) was defined as ≥ 30 mmHg and a high LVOT peak velocity as > 1.0 m/s. Given that patient age may affect the results, we conducted subset analyses of patients aged ≥ 75 years at the time of surgery.

### Surgical procedures

Surgery was performed using median sternotomy and standard cardiopulmonary bypass techniques. Decisions for myectomy were made by the operating surgeons based on preoperative diagnosis of HCM or TTE findings, or intraoperative observations of SAM or septal protrusion into the LVOT.

### Histopathological analysis

The resected septa were sent to the pathology core facility and preserved in paraffin-embedded tissue blocks. Several of these blocks were randomly chosen and cut into 4 μm-thick slices. Masson's trichrome staining was used to identify fibrotic tissues. To quantify the degree of fibrosis, digital images of the stained slices were acquired using a multifunctional color laser machine, Bizhub c368 (Konica Minolta, Tokyo, Japan). Subsequently, the blue stain, indicative of collagenous connective tissue, was recognized and overlaid green using an image analysis software, KS400 (Carl Zeiss AG, Oberkochen, Germany). Finally, the software was programmed to calculate the ratio of green area to purple tissue background to quantify the degree of fibrosis.

### Statistical analysis

Statistical analyses were performed using R 4.0.2 (R Foundation for Statistical Computing, Vienna, Austria), EZR^23)^, and Microsoft Excel 2016 (Microsoft Corporation, Redmond, Washington, USA). Continuous numerical and categorical variables are presented as mean ± standard deviation and number (%), respectively. Discrete numerical, non-normally distributed variables are presented as median (minimum, maximum). Continuous numerical and categorical variables were compared using Student's *t*-test and Fisher's exact test, respectively, between unpaired groups, and using paired Student's *t*-test and McNemar's test with continuity correction, respectively, between paired groups. Survival rate was calculated using Kaplan–Meier analysis. A *P*-value < 0.05 was considered statistically significant.

## Results

### Patient demographics and baseline clinical characteristics

Patient demographics and baseline clinical characteristics are summarized in Table 1. Forty-three patients (6.4%) underwent myectomy with SAVR. The average age at surgery and the proportion of female patients were significantly higher in the myectomy group than in the AVR group (*P* < 0.05 and *P* < 0.001, respectively). Given the female predominance, the average body surface area was significantly smaller in the myectomy group than in the AVR group (*P* < 0.05). Degenerative etiology was most frequent in both the groups. The elderly subset analysis also revealed strong female predominance in the myectomy group (Table 2).

**Table 1:** Baseline clinical characteristics of patients with severe aortic stenosis undergoing aortic valve replacement

Characteristics	All patients (n = 671)	AVR (n = 628)	Myectomy (n = 43)	*P*-value
Age [years]	72.8 ± 9.3	72.5 ± 9.4	76.2 ± 6.6	< 0.05
Sex, n (%)				
Female	334 (49.8)	301 (47.9)	33 (76.7)	< 0.001
Male	337 (50.2)	327 (52.1)	10 (23.3)	
Body mass index [kg/m^2^]	22.9 ± 3.6	22.8 ± 3.5	23.6 ± 3.9	0.163
Body surface area [m^2^]	1.57 ± 0.19	1.58 ± 0.19	1.51 ± 0.17	< 0.05
Etiology, n (%)				
Degenerative	481 (71.7)	442 (70.4)	39 (90.7)	< 0.05
Bicuspid valve	133 (19.8)	130 (20.7)	3 (7.0)	
Rheumatic	28 (4.2)	28 (4.5)	0 (0.0)	
Artificial valve dysfunction	29 (4.3)	28 (4.5)	1 (2.3)	

Continuous numerical and categorical variables are presented as mean ± standard deviation and number (%), respectively, and were compared using Student's *t*-test and Fisher's exact test, respectively, between the groups. AVR, aortic valve replacement.

**Table 2:** Baseline clinical characteristics of elderly subset with severe aortic stenosis undergoing aortic valve replacement (aged ≥ 75 years)

Characteristics	All patients (n = 333)	AVR (n = 309)	Myectomy (n = 24)	*P*-value
Age [years]	79.4 ± 3.3	79.3 ± 3.2	80.8 ± 4.0	< 0.05
Sex, n (%)				
Female	171 (51.4)	149 (48.2)	22 (91.7)	< 0.001
Male	162 (48.6)	160 (51.8)	2 (83)	
Body mass index [kg/m^2^]	22.7 ± 3.1	22.8 ± 3.2	22.5 ± 2.8	0.733
Body surface area [m^2^]	1.54 ± 0.17	1.55 ± 0.17	1.43 ± 0.14	< 0.01
Etiology, n (%)				
Degenerative	287 (86.2)	263 (85.1)	24 (100.0)	0.391
Bicuspid valve	27 (8.1)	27 (8.7)	0 (0.0)	
Rheumatic	12 (3.6)	12 (3.9)	0 (0.0)	
Artificial valve dysfunction	7 (2.1)	7 (2.3)	0 (0.0)	

Continuous numerical and categorical variables are presented as mean ± standard deviation and number (%), respectively, and were compared using Student's *t*-test and Fisher's exact test, respectively, between the groups. AVR, aortic valve replacement.

### Baseline echocardiography

Baseline TTE parameters are presented in Table 3. Compared with the normal reference values, the group of all patients had a significantly thicker IVS, PW, and RWT; and a significantly larger LVM and LVMI, as marked with asterisk signs (all *P* < 0.001). In contrast, only the myectomy group had a smaller left ventricular dimension during diastole (LVDd) and systole (LVDs), as marked with hash signs (both *P* < 0.001), and a higher left ventricular ejection fraction (LVEF) (*P* < 0.001). In short, the patients undergoing SAVR for sAS had concentric LVH, and the myectomy group additionally exhibited a smaller LV size and stronger systolic function than the normal references.

**Table 3:** Baseline echocardiographic findings of patients with severe aortic stenosis undergoing aortic valve replacement

Characteristics	All patients (n = 671)	AVR (n = 628)	Myectomy (n = 43)	*P*-value
LVDd [mm]	47.3 ± 6.6	47.6 ± 6.6	43.0 ± 5.2^###^	< 0.001
LVDs [mm]	30.8 ± 7.4	31.2 ± 7.4**	25.3 ± 3.9^###^	< 0.001
IVS [mm]	11.4 ± 1.8***	11.3 ± 1.8***	12.5 ± 1.7***	< 0.001
IVS ≥ 15 [mm], n (%)	29 (4.3)	25 (4.0)	4 (9.3)	0.107
PW [mm]	11.3 ± 1.7***	11.2 ± 1.7***	12.1 ± 1.4***	< 0.01
PW ≥ 15 [mm], n (%)	21 (3.1)	19 (3.0)	2 (4.7)	0.638
IVS/PW > 1.3, n (%)	7 (1.0)	6 (1.0)	1 (2.3)	0.372
RWT	0.49 ± 0.10***	0.48 ± 0.10***	0.57 ± 0.10***	< 0.001
LVM [g]	201.3 ± 62.1***	201.8 ± 62.8***	194.2 ± 51.0***	0.441
LVMI [g/m^2^]	127.8 ± 35.3***	127.7 ± 35.4***	129.7 ± 33.6***	0.719
LVEF [%]	63.6 ± 11.9	63.1 ± 12.0	71.1 ± 7.4***	< 0.001
AVA [cm^2^]	0.74 ± 0.19	0.73 ± 0.19	0.84 ± 0.25	< 0.001
AVAI [cm^2^/m^2^]	0.47 ± 0.13	0.47 ± 0.12	0.56 ± 0.16	< 0.001
AV mPG [mmHg]	45.2 ± 17.8	44.8 ± 17.8	51.2 ± 16.9	< 0.05
AV peak velocity [m/s]	4.4 ± 0.8	4.3 ± 0.8	4.7 ± 0.8	< 0.01
AV cusp, n (%)				
Artificial	29 (4.3)	28 (4.5)	1 (2.3)	< 0.05
Bicuspid	133 (19.8)	130 (20.7)	3 (7.0)	
Tricuspid	468 (69.7)	435 (69.3)	33 (76.7)	
Quadricuspid	1 (0.1)	1 (0.2)	0 (0.0)	
Unknown	40 (6.0)	34 (5.4)	6 (14.0)	

Continuous numerical and categorical variables are presented as mean ± standard deviation and number (%), respectively, and were compared using Student's *t*-test and Fisher's exact test, respectively, between the groups. Some variables were compared with their normal reference values using paired Student's *t*-test, as described in ‘Patients and Methods'. *P < 0.05, **P < 0.01, ***P < 0.001 larger than the reference value. ^#^*P* < 0.05, ^##^*P* < 0.01, ^###^*P* < 0.001 smaller than the reference value. AVR, aortic valve replacement; NA, not applicable or unavailable; LVDd, left ventricular dimension during diastole; LVDs, left ventricular dimension during systole; IVS, interventricular septum; PW, posterior wall; RWT, relative wall thickness; LVM, left ventricular mass; LVMI, left ventricular mass index; LVEF, left ventricular ejection fraction; AVA, aortic valve area; AVAI, aortic valve area index; AV, aortic valve; mPG, mean pressure gradient.

Compared with the AVR group, the myectomy group had a smaller LVDd and LVDs (both *P* < 0.001); a thicker IVS (*P* < 0.001), PW (*P* < 0.01), and RWT (*P* < 0.001); a higher LVEF (*P* < 0.001); and a larger AVA, AVA index (AVAI) (both *P* < 0.001), AV mPG (*P* < 0.05), and AV peak velocity (*P* < 0.01). Therefore, compared with the AVR counterpart, the myectomy group had an even smaller but more hypertrophic LV, an even higher LVEF, and more severe AS in terms of AV mPG and AV peak velocity. For the number of AV cusps, tricuspid was most frequent in both the groups.

The elderly subset analysis revealed comparable results (Table 4). However, statistical significance of larger AV mPG and AV peak velocity in the myectomy group diminished, possibly due to the small sample size (n = 24).

**Table 4:** Baseline echocardiographic findings of elderly subset with severe aortic stenosis undergoing aortic valve replacement (aged ≥ 75 years)

Characteristics	All patients (n = 333)	AVR (n = 309)	Myectomy (n = 24)	*P*-value
LVDd [mm]	46.6 ± 6.1^##^	47.0 ± 6.0^#^	41.3 ± 4.9^##^	< 0.001
LVDs [mm]	30.1 ± 7.0	30.6 ± 7.0	24.4 ± 4.1^###^	< 0.001
IVS [mm]	11.3 ± 1.8***	11.2 ± 1.7***	12.4 ± 1.8***	< 0.01
IVS ≥ 15 [mm], n (%)	13 (3.9)	10 (3.2)	3 (12.5)	0.058
PW [mm]	11.3 ± 1.6***	11.2 ± 1.6***	12.2 ± 1.6***	< 0.01
PW ≥ 15 [mm], n (%)	7 (2.1)	6 (1.9)	1 (4.2)	0.411
IVS/PW > 1.3, n (%)	4 (1.2)	3 (1.0)	1 (4.2)	0.260
RWT	0.49 ± 0.10***	0.48 ± 0.09***	0.60 ± 0.12***	< 0.001
LVM [g]	195.5 ± 55.9***	196.6 ± 57.1***	180.8 ± 35.5***	0.181
LVMI [g/m^2^]	126.9 ± 33.1***	126.9 ± 33.4***	127.8 ± 30.2***	0.895
LVEF [%]	64.3 ± 11.9	63.7 ± 12.0	72.1 ± 6.6***	< 0.01
AVA [cm^2^]	0.72 ± 0.17	0.71 ± 0.17	0.81 ± 0.21	< 0.01
AVAI [cm^2^/m^2^]	0.47 ± 0.12	0.46 ± 0.11	0.57 ± 0.14	< 0.001
AV mPG [mmHg]	44.2 ± 16.9	43.8 ± 16.9	49.1 ± 16.2	0.140
AV peak velocity [m/s]	4.3 ± 0.8	4.3 ± 0.8	4.6 ± 0.8	0.073
AV cusp, n (%)				
Artificial	7 (2.1)	7 (2.3)	0 (0.0)	0.482
Bicuspid	27 (8.1)	27 (8.7)	0 (0.0)	
Tricuspid	284 (85.3)	261 (84.5)	23 (95.8)	
Quadricuspid	0 (0.0)	0 (0.0)	0 (0.0)	
Unknown	15 (4.5)	14 (4.5)	1 (4.2)	

Continuous numerical and categorical variables are presented as mean ± standard deviation and number (%), respectively, and were compared using Student's *t*-test and Fisher's exact test, respectively, between the groups. Some variables were compared with their normal reference values using paired Student's *t*-test, as described in ‘Patients and Methods’. *P < 0.05, **P < 0.01, ***P < 0.001 larger than the reference value. ^#^*P* < 0.05, ^##^*P* < 0.01, ^###^*P* < 0.001 smaller than the reference value. AVR, aortic valve replacement; NA, not applicable or unavailable; LVDd, left ventricular dimension during diastole; LVDs, left ventricular dimension during systole; IVS, interventricular septum; PW, posterior wall; RWT, relative wall thickness; LVM, left ventricular mass; LVMI, left ventricular mass index; LVEF, left ventricular ejection fraction; AVA, aortic valve area; AVAI, aortic valve area index; AV, aortic valve; mPG, mean pressure gradient.

Compared with the normal reference values, baseline TTE parameters from each gender subgroup within the myectomy group exhibited the same significant trends as the whole myectomy group (smaller LVDd and LVDs, thicker IVS, PW, and RWT, and larger LVM and LVMI, all *P* < 0.05 with some < 0.01 or < 0.001), but only the female group had a higher LVEF (paired Student's *t*-test, *P* < 0.001). Baseline TTE parameters from female and male subgroups within the myectomy group were similar to each other (LVDd, LVDs, IVS, PW, RWT, LVM, LVMI, AVA, AVAI, AV mPG, AV peak velocity, all *P* ≥ 0.05), except LVEF (unpaired Student's *t*-test, female 73% vs. male 66%, *P* < 0.05).

### Comorbidities

Comorbidities of patients in the myectomy group are listed in Table 5. Hypertension was most frequently observed. The Charlson Comorbidity Index score had a median of 4 (range, 2 to 9). Functional status was mostly New York Heart Association functional classification II (67%). The data for the elderly subset were comparable.

**Table 5:** Comorbidities of patients with severe aortic stenosis undergoing aortic valve replacement with concomitant myectomy

Characteristics	Myectomy (n = 43)	Subset ≥ 75 yo (n = 24)
Comorbidities, n (%)		
Hypertension	31 (72.1)	19 (79.2)
Dyslipidemia	28 (65.1)	16 (66.7)
Diabetes mellitus	10 (23.3)	4 (16.7)
Cerebrovascular disease	5 (11.6)	4 (16.7)
Peripheral vascular disease	4 (9.3)	4 (16.7)
Dialysis	2 (4.7)	0 (0.0)
Connective tissue disease	1 (2.3)	1 (4.2)
COPD	1 (2.3)	0 (0.0)
Hypertrophic cardiomyopathy	1 (2.3)	0 (0.0)
Liver dysfunction	1 (2.3)	0 (0.0)
Prior myocardial infarction	1 (2.3)	0 (0.0)
Charlson Comorbidity Index, median (range)	4 (2, 9)	5 (3, 9)
Symptoms, n (%)		
NYHA I	9 (20.9)	4 (16.7)
NYHA II	29 (67.4)	16 (66.7)
NYHA III	4 (9.3)	3 (12.5)
NYHA IV	1 (2.3)	1 (4.2)

Discrete numerical, non-normally distributed variables and categorical variables are presented as median (minimum, maximum) and number (%), respectively. yo, years old; COPD, chronic obstructive pulmonary disease; NYHA, New York Heart Association.

### Operative details

Operative details of patients in the myectomy group are presented in Table 6. The procedural time of the myectomy group was similar to that of the AVR group (242 minutes vs. 269 minutes, *P* = 0.09). Most of the implanted valves were of bioprosthetic type and 19 or 21 mm in diameter. Left atrial appendage resection was the most frequent concomitant procedure performed with SAVR in addition to myectomy. The data for the elderly subset were also comparable. Of note, 2 of the 5 concomitant mitral valve plasty procedures were added intraoperatively based on the new onset of SAM (4.7%).

**Table 6:** Operative details of patients with severe aortic stenosis undergoing aortic valve replacement with concomitant myectomy

Characteristics	Myectomy (n = 43)	Subset ≥ 75 yo (n = 24)
Procedural time [minutes]	241.8 ± 94.2	216.4 ± 66.4
Cardiopulmonary bypass duration [minutes]	115.0 ± 44.3	99.7 ± 31.9
Aortic cross-clamp duration [minutes]	91.8 ± 38.3	79.8 ± 27.0
Valve prosthesis type, n (%)		
Bioprosthesis	42 (97.7)	24 (100.0)
Mechanical	1 (2.3)	0 (0.0)
Valve prosthesis size, n (%)		
19 [mm]	15 (34.9)	10 (41.7)
21 [mm]	15 (34.9)	8 (33.3)
23 [mm]	10 (23.3)	6 (25.0)
25 [mm]	3 (7.0)	0 (0.0)
Concomitant procedures, n (%)		
LAA resection	41 (95.3)	23 (95.8)
Chordal cutting	8 (18.6)	3 (12.5)
CABG	6 (14.0)	3 (12.5)
MVP	5^a^ (11.6)	1 (4.2)
TAP	2 (4.7)	2 (8.3)
MVR	1 (2.3)	1 (4.2)
Repeat cardiac procedure, n (%)	1 (2.3)	0 (0.0)

Continuous numerical and categorical variables are presented as mean ± standard deviation and number (%), respectively. ^a^ Two of the 5 MVP procedures were added intraoperatively based on the new onset of SAM (4.7%); one observed before and the other after weaning from the cardiopulmonary bypass. yo, years old; LAA, left atrial appendage; CABG, coronary artery bypass grafting; MVP, mitral valve plasty; TAP, tricuspid annuloplasty; MVR, mitral valve replacement.

### Changes in echocardiography

Table 7 presents the TTE parameters of patients in the myectomy group in preoperative and two postoperative periods. Immediately postoperative and 1-year postoperative TTE were obtained on the median postoperative day (POD) 7 (range, 1 to 88) and POD 366 (range, 193 to 534), respectively. Surgery significantly reduced the IVS, PW, and RWT after 1 year, although they remained thicker than the reference values (*P* < 0.001). Similarly, surgery significantly reduced the LVM and LVMI after 1 year, although these values remained larger than the reference values (*P* < 0.001). In short, concentric LVH improved over time, but not completely to normal. The frequencies of IVS ≥ 15 mm, PW ≥ 15 mm, and IVS/PW > 1.3 had decreased to zero at 1 year. The presence of sigmoid septum or SAM was recorded only when echo technicians were able to identify them, and the frequencies of these were not significantly altered at 1 year. LVOT hemodynamic parameters were not recorded for all patients for the same reason, and yet, comparison using available data (n = 16) revealed a moderate decrease in LVOT pPG (*P* = 0.172) and a significant decrease in LVOT peak velocity (*P* < 0.05) at 1 year. Therefore, parameters indicative of LVOTO improved after surgery. The LVEF decreased immediately after surgery but not at 1 year and remained higher than the reference value at 1 year (*P* < 0.05). As a result of SAVR, the smaller AVA and AVAI and larger AV mPG and AV peak velocity in sAS improved immediately after surgery and remained stable after 1 year.

**Table 7:** Echocardiographic changes in patients with severe aortic stenosis undergoing aortic valve replacement with concomitant myectomy

Characteristics	Preoperative (n = 43)	Immediately postoperative (n = 43)	*P*-value	1 year postoperative (n = 25)	*P*-value
LVDd [mm]	43.0 ± 5.2^###^	41.1 ± 4.2^###^	< 0.05	43.2 ± 3.7^###^	0.964
LVDs [mm]	25.3 ± 3.9^###^	25.8 ± 2.9^###^	0.410	26.3 ± 3.0^###^	0.172
IVS [mm]	12.5 ± 1.7***	12.3 ± 2.1***	0.409	11.0 ± 1.2***	< 0.001
≥ 15 [mm], n (%)	4 (9.3)	4 (9.3)	1.00	0 (0.0)	NA
PW [mm]	12.1 ± 1.4***	11.7 ± 1.6***	< 0.05	10.9 ± 0.9***	< 0.001
≥ 15 [mm], n (%)	2 (4.7)	1 (2.3)	1.00	0 (0.0)	NA
IVS/PW > 1.3, n (%)	1 (2.3)	1 (2.3)	NA	0 (0.0)	NA
RWT	0.57 ± 0.10***	0.57 ± 0.11***	0.931	0.51 ± 0.07***	< 0.05
Sigmoid septum, n (%)	11 (25.6)	1 (2.3)	< 0.01	4 (16)	0.450
SAM, n (%)	8 (18.6)	5 (11.6)	0.450	1 (4)	0.248
LVOT pPG [mmHg]	18.7 ± 30.1	12.1 ± 13.6^a^	0.163	7.5 ± 11.8^b^	0.172
≥ 30 [mmHg], n (%)	5 (11.6)	3 (13.0)^a^	1.00	1 (6.3)^b^	NA
LVOT peak velocity [m/s]	1.92 ± 1.34	1.65 ± 0.87^a^	0.251	1.2 ± 0.67^b^	< 0.05
> 1.0 [m/s], n (%)	23 (53.5)	17 (73.9)^a^	1.00	10 (39)^b^	1.00
LVM [g]	194.2 ± 51.0***	173.8 ± 41.5***	< 0.001	164.9 ± 28.6***	< 0.01
LVMI [g/m^2^]	129.7 ± 33.6***	116.9 ± 27.4***	< 0.001	111.2 ± 24.5***	< 0.01
LVEF [%]	71.1 ± 7.4***	66.8 ± 7.4**	< 0.01	67.6 ± 8.1*	0.081
AVA [cm^2^]	0.84 ± 0.25	1.82 ± 0.63	< 0.001	1.84 ± 0.56	< 0.001
AVAI [cm^2^/m^2^]	0.56 ± 0.16	1.22 ± 0.39	< 0.001	1.22 ± 0.31	< 0.001
AV mPG [mmHg]	51.2 ± 16.9	11.7 ± 4.8	< 0.001	9.8 ± 3.8	< 0.001
AV peak velocity [m/s]	4.7 ± 0.8	2.4 ± 0.5	< 0.001	2.2 ± 0.4	< 0.001

Continuous numerical and categorical variables are presented as mean ± standard deviation and number (%), respectively, and were compared using paired Student's *t*-test and McNemar's test with continuity correction, respectively, between the groups (preoperative vs. immediately postoperative, and preoperative vs. 1 year postoperative). Some variables were compared with their normal reference values using paired Student's *t*-test, as described in ‘Patients and Methods’. *P < 0.05, **P < 0.01, ***P < 0.001 larger than the reference value. ^#^*P* < 0.05, ^##^*P* < 0.01, ^###^*P* < 0.001 smaller than the reference value. ^a^ n = 23. ^b^ n = 16. LVDd, left ventricular dimension during diastole; LVDs, left ventricular dimension during systole; IVS, interventricular septum; PW, posterior wall; RWT, relative wall thickness; SAM, systolic anterior motion of the mitral valve; LVOT, left ventricle outflow tract; pPG, peak pressure gradient; LVM, left ventricular mass; LVMI, left ventricular mass index; LVEF, left ventricular ejection fraction; AVA, aortic valve area; AVAI, aortic valve area index; AV, aortic valve; mPG, mean pressure gradient.

The elderly subset analysis revealed comparable results (Table 8). However, statistical significance of changes in the RWT and LVOT peak velocity at 1 year diminished, possibly due to the small sample sizes again (n = 12 and n = 7, respectively).

**Table 8:** Echocardiographic changes in elderly subset with severe aortic stenosis undergoing aortic valve replacement with concomitant myectomy (aged ≥ 75 years)

Characteristics	Preoperative (n = 24)	Immediately postoperative (n = 24)	*P*-value	1 year postoperative (n = 12)	*P*-value
LVDd [mm]	41.3 ± 4.9^##^	39.8 ± 4.0^###^	0.115	42.3 ± 4.1^#^	0.377
LVDs [mm]	24.4 ± 4.1^###^	25.2 ± 2.9^###^	0.360	25.9 ± 2.6^#^	0.148
IVS [mm]	12.4 ± 1.8***	12.4 ± 2.1***	0.935	11.1 ± 1.0***	< 0.05
≥ 15 [mm], n (%)	3 (12.5)	2 (8.3)	1.00	0 (0.0)	NA
PW [mm]	12.2 ± 1.6***	11.4 ± 1.2***	< 0.05	11.1 ± 0.8***	< 0.01
≥ 15 [mm], n (%)	1 (4.2)	0 (0.0)	NA	0 (0.0)	NA
IVS/PW > 1.3, n (%)	1 (4.2)	1 (4.2)	NA	0 (0.0)	NA
RWT	0.60 ± 0.12***	0.58 ± 0.09***	0.378	0.53 ± 0.07***	0.060
Sigmoid septum, n (%)	7 (29.2)	0 (0.0)	NA	2 (16.7)	NA
SAM, n (%)	4 (16.7)	3 (12.5)	1.00	1 (8.3)	1.00
LVOT pPG [mmHg]	18.0 ± 30.8	10.7 ± 14.5^a^	0.177	12.1 ± 17.3^b^	0.360
≥ 30 [mmHg], n (%)	2 (8.3)	1 (4.2)^a^	1.00	1 (8.3)^b^	NA
LVOT peak velocity [m/s]	1.58 ± 1.24	1.41 ± 0.87^a^	0.358	1.54 ± 0.94^b^	0.511
> 1.0 [m/s], n (%)	7 (29.2)	8 (33.3)^a^	1.00	6 (50)^b^	0.480
LVM [g]	180.8 ± 35.5***	162.9 ± 30.1***	< 0.05	161.2 ± 27.9***	< 0.01
LVMI [g/m^2^]	127.8 ± 30.2***	115.3 ± 22.7***	< 0.05	114.6 ±26.7***	< 0.05
LVEF [%]	72.1 ± 6.6***	68.1 ± 7.1**	0.053	69.0 ± 6.7*	0.111
AVA [cm^2^]	0.81 ± 0.21	1.75 ± 0.63	< 0.001	1.80 ± 0.56	< 0.001
AVAI [cm^2^/m^2^]	0.57 ± 0.14	1.23 ± 0.44	< 0.001	1.25 ± 0.32	< 0.001
AV mPG [mmHg]	49.1 ± 16.2	12.3 ± 5.5	< 0.001	10.9 ± 4.1	< 0.001
AV peak velocity [m/s]	4.6 ± 0.8	2.4 ± 0.5	< 0.001	2.3 ± 0.4	< 0.001

Continuous numerical and categorical variables are presented as mean ± standard deviation and number (%), respectively, and were compared using paired Student's *t*-test and McNemar's test with continuity correction, respectively, between the groups (preoperative vs. immediately postoperative, and preoperative vs. 1 year postoperative). Some variables were compared with their normal reference values using paired Student's *t*-test, as described in ‘Patients and Methods’. *P < 0.05, **P < 0.01, ***P < 0.001 larger than the reference value. ^#^*P* < 0.05, ^##^*P* < 0.01, ^###^*P* < 0.001 smaller than the reference value. ^a^ n = 11. ^b^ n = 7. LVDd, left ventricular dimension during diastole; LVDs, left ventricular dimension during systole; IVS, interventricular septum; PW, posterior wall; RWT, relative wall thickness; SAM, systolic anterior motion of the mitral valve; LVOT, left ventricle outflow tract; pPG, peak pressure gradient; LVM, left ventricular mass; LVMI, left ventricular mass index; LVEF, left ventricular ejection fraction; AVA, aortic valve area; AVAI, aortic valve area index; AV, aortic valve; mPG, mean pressure gradient.

### Complications and prognosis

Complications and prognosis of patients in the myectomy group are summarized in Table 9. Intraoperative or 30-day all-cause mortality was not observed. The median stay in the intensive care unit or in the hospital after surgery were 1 day (range, 1 to 14) and 16 days (range, 7 to 77), respectively. Most of the discharge disposition locations were home (86%). There was one in-hospital mortality (2.3%). A 74-year-old woman, with the Charlson Comorbidity Index of 5, died on POD 73 from worsening mitral regurgitation, pneumonia, and sepsis. As non-lethal complications, a complete atrioventricular block requiring a new permanent pacemaker (PPM) implantation was found in three patients, and a ventricular septal defect at the myectomy site, thromboembolic stroke on POD 10, and DeBakey type IIIb aortic dissection on POD 5 in one patient each. The median follow-up interval was 1,038 days (range, 12 to 2,577).

**Table 9:** Complications and prognosis of patients with severe aortic stenosis undergoing aortic valve replacement with concomitant myectomy

Characteristics	Myectomy (n = 43)	Subset ≥ 75 yo (n = 24)
30-day all-cause mortality, n (%)	0 (0.0)	0 (0.0)
Days in ICU, median (range)	1 (1, 14)	1 (1, 9)
Days until discharge, median (range)	16 (7, 77)	16 (7, 36)
Disposition, n (%)		
Home	37 (86.0)	20 (83.3)
Transfer	5 (11.6)	4 (16.7)
NA^a^	1 (2.3)	0 (0.0)
Complications, n (%)		
Complete atrioventricular block	3 (7.0)	2 (8.3)
Ventricular septal defect	1 (2.3)	1 (4.2)
Stroke	1 (2.3)	1 (4.2)
Aortic dissection	1 (2.3)	0 (0.0)

Discrete numerical, non-normally distributed variables and categorical variables are presented as median (minimum, maximum) and number (%), respectively. ^a^ One in-hospital mortality. yo, years old; ICU, intensive care unit; NA, not applicable.

**Figure 1:**
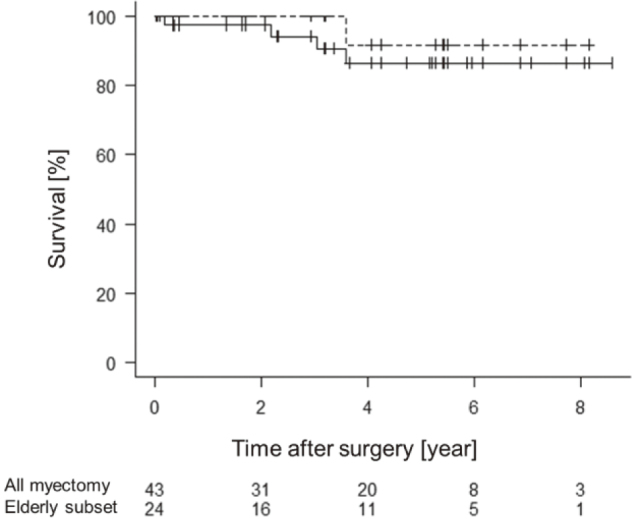
Kaplan–Meier plot of the myectomy group and its elderly subset (aged ≥ 75 years). Solid line, myectomy group; dotted line, elderly subset. The survival rates at postoperative year 1, 3, 5, and 7 were 97%, 94%, 86%, and 86%, respectively, in the myectomy group, whereas they were 100%, 100%, 92%, and 92% in the elderly subset.

Kaplan–Meier plot of the myectomy group is displayed in Figure 1. The survival rates at postoperative year 1, 3, 5, and 7 were 97%, 94%, 86%, and 86%, respectively. The data for the elderly subset were comparable.

### Fibrosis in resected tissues

**Figure 2:**
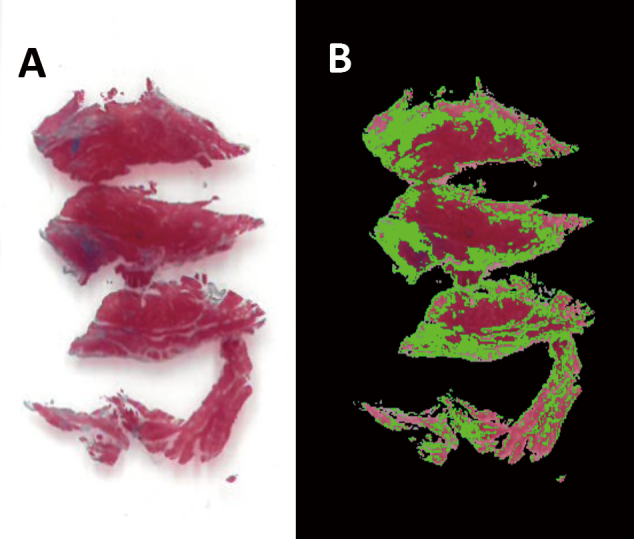
Fibrosis in resected tissue. (A) Masson's trichrome staining of the resected interventricular septum in patients with severe aortic stenosis undergoing aortic valve replacement. (B) Same histopathological slide analyzed for the degree of fibrosis.

Figure 2A shows histological preparation of resected IVS. Figure 2B shows image processing using the image analysis software. Spotty fibrotic tissues were clearly visible on gross examination of the stained slides (blue in Figure 2A). However, the degree of fibrosis calculated using the software varied greatly across tissue blocks (data not shown), and upon microscopic observation at the pathology core facility, most were observed to have slight to moderate fibrosis.

## Discussion

**Figure 3:**
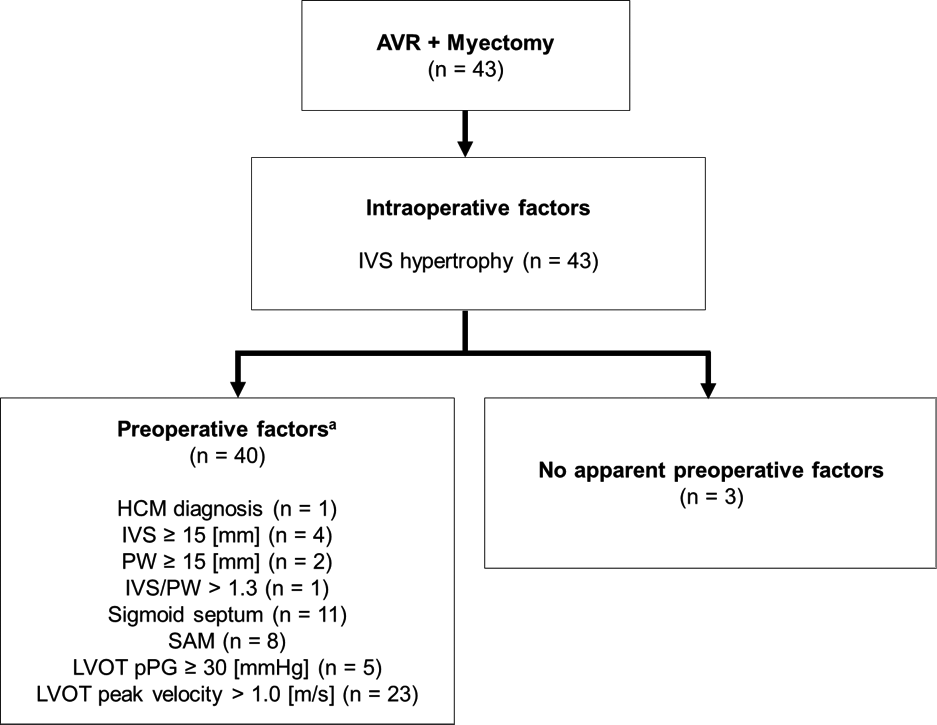
Risk factors for LVOTO in patients with severe aortic stenosis undergoing aortic valve replacement with concomitant myectomy. ^a^ Some patients had multiple factors. AVR, aortic valve replacement; IVS, interventricular septum; HCM, hypertrophic cardiomyopathy; PW, posterior wall; SAM, systolic anterior motion of the mitral valve; LVOT, left ventricular outflow tract; pPG, peak pressure gradient.

The patients' demographics, such as female predominance (77%) and older age at surgery (76 years old), as well as the etiology (mainly degenerative) and comorbidity (hypertension most common) were in line with the previous report on patients with sAS in need of myectomy^17)^. These observations were replicated across the Pacific despite large sociomedical and genetic differences, although the female predominance may in part stem from longer lifespan of women worldwide^24)^. Echocardiographically, patients in the myectomy group were further characterized by a small-in-size, yet extra- hypertrophic and extra-hypercontractile heart and more severe AS compared with the AVR counterpart. This may be explained by bidirectional causality between sAS and LVH; sAS causes chronically raised afterload, which stimulates compensatory LVH. Inversely, LVH in a compensatory phase causes a higher flow across the AV, which leads to more severe form of AS. Myectomy with SAVR relieves them both. Figure 3 shows retrospectively analyzed factors predisposing to LVOTO. Most of the patients in the myectomy group (93%) had at least one of these factors. We presume predictive values of these factors, and await further studies for confirmation.

With regard to the effectiveness of myectomy, this study showed that myectomy with SAVR effectively improved concentric LVH and LVOTO. AVR alone reduces the PW and LVMI in sAS^25)^; therefore, how the addition of myectomy affected the improvement of concentric LVH is unclear. In contrast, the improvement of LVOTO may be reasonably attributable to myectomy rather than AVR, because myectomy has been known to relieve LVOTO^4)^, and conversely, AVR has been known to unmask it^15, 16)^.

Regarding procedural safety, some authors recommend alcohol septal ablation for elderly patients with HCM as an alternative to myectomy. However, survival rates and complication profiles were not particularly worse for our patients in the myectomy group, including the elderly subset. Thirty-day all-cause mortality was not observed, and although one in-hospital mortality was observed, the risk (2.3%) could have been overestimated due to the small sample size (n = 43). Moreover, the long-term survival was comparable to that reported previously^17)^. The frequency of new PPM implantation (7.0%) was similar to that in SAVR alone (8.5%)^26)^, despite the possibility of either SAVR or myectomy affecting the conduction system. The frequency of iatrogenic ventricular septal defect, a rare but important complication (2.3%), was slightly greater than in patients with HCM after myectomy^27)^, but its significance remains unclear due to the small sample size. Overall, this study showed that myectomy with SAVR was relatively safe.

Histologically, myocardial fibrosis (MF) has been found in various diseases including hypertension and HCM^28)^. Therefore, we had expected significant MF in resected IVS. Instead, pathology reports indicated slight to moderate MF. In HCM, MF is common^29)^ and strongly associated with the occurrence of systolic dysfunction^30)^, ventricular tachyarrhythmia^31)^, and major adverse events^32)^. In AS, MF has a significant negative correlation with symptomatic improvements^33)^, systolic function^34)^, and long-term survival after SAVR^35)^. Therefore, the hypercontractile heart of patients in the myectomy group here actually corresponds with mild MF as demonstrated. The decisions for surgery were perhaps made sufficiently early, because they were made before MF became significant enough to cause hemodynamic decompensation.

Lastly, to further investigate effectiveness of myectomy, a randomized controlled trial should be performed where LVOTO risk factors and severity of LVOTO are matched between myectomy and non-myectomy groups. However, difficulties in such studies are multifold. As already known, LVOTO is an extremely heterogeneous disease state, and various associated findings have been reported but universally accepted risk factors are yet to be defined. Intraoperative decisions for myectomy are also yet to be standardized. Thus, it would be technically challenging to prepare appropriate groups for comparison while controlling all possible confounding factors. In more practical terms, a patient's assignment to non-myectomy group would create an ethical dilemma for the operating surgeons as they leave hypertrophied IVS untouched, knowing the high risk of postoperative LVOTO. On the whole, LVOTO risk factors require additional investigations. For more comprehensive risk assessment, additional use of operator-independent imaging modalities may help.

## Limitations

The biggest limitation of this study was that it was a retrospective single-arm study because of the difficulty in controlling confounding factors as described above. It was conducted at a single center; therefore, extrapolation of the results obtained here to wider patient populations may require further confirmation.

## Conclusions

Myectomy can be safely performed with SAVR for sAS with LVOTO, even in elderly patients (≥ 75 years), and is effective in improving concentric LVH and LVOTO. To detect surgically amenable entities contributing to LVOTO and avoid “suicide LV,” we first propose that clinicians should be aware of such entities in various clinical situations with hemodynamic instability. Second, elderly female patients with relatively more severe AS and a small yet extra-hypertrophic and extra-hypercontractile heart are of particular interest. For these patients, a comprehensive assessment of LVOTO risk factors would be necessary. It is critical to acknowledge and identify such a patient group because, despite its invasiveness, SAVR may be potentially more beneficial for them by allowing direct observation of LVOT and ancillary myectomy.

## Funding

This work was supported by the Department of Cardiovascular Surgery, Juntendo University Graduate School of Medicine. The authors have received no external financial support for this study.

## Author contributions

AU collected, analyzed, and interpreted the data. AU also processed the histological slices and is the main author of this manuscript. SM, TK, and MT supervised and reviewed the process. All authors read and approved the final manuscript.

## Conflicts of interest statement

The authors declare that they have no conflicts of interest.
